# Radial neck fracture in children: anatomic and functional results of Metaizeau technique

**DOI:** 10.11604/pamj.2020.36.144.22971

**Published:** 2020-06-30

**Authors:** Ahmed Trabelsi, Mohamed Ali Khalifa, Rim Brahem, Mehdi Jedidi, Karim Bouattour, Walid Osman, Mohamed Laziz Ben Ayeche

**Affiliations:** 1 Department of Orthopedic Surgery, Sahloul University Hospital, Sousse, Tunisia,; 2 Department of Rehabilitation Medicine, Sahloul University Hospital, Sousse, Tunisia

**Keywords:** Metaizeau, radial Neck, fracture, child

## Abstract

Fractures of the radial neck accounts for 1% of all childhood fractures and 5% to 10% of childhood traumatic lesions involving the elbow. Intramedullary percutaneous nail reduction (Metaizeau technique) is considered the most effective surgical technique. The purpose of this study was to identify the main clinical features of radial neck fracture in children and to evaluate the anatomical and functional results of the Metaizeau technique. In this retrospective study, we evaluated 22 patients under the age of 16 who were treated for radial neck fracture at the orthopedic and trauma surgery department of Sahloul University Hospital in Sousse over a period of 16 years from January 2001 to April 2017. Authors used Metaizeau classification. Functional results were evaluated by Mayo elbow performance score (MEPS) and the radiological evaluation was based on standard images with measurement of the residual rocker. The average age was 8.6 years (5-13 years). Seven fracture were grade III injuries and three grade IV. In the immediate postoperative period, radiological measurements showed a residual rocker less than 20° in 86.3% and more than 20° in 13.7% of cases. At an average follow-up of 13 months and a half, the MEPS score was excellent and good for 17 patients. Four types of complications were found: necrosis of the radial head in 1 case, pseudarthrosis in 1 case, periarticular calcification in 2 cases and stiff-ness of the elbow in 3 cases. Despite the small number of patients in our series, we believe that the elastic stable intramedullary pinning according to the Metaizeau technique is the treatment of choice for displaced radial neck fractures in children.

## Introduction

Radial neck fractures are relatively rare in children, accounting for 1% of all fractures in children and 5% to 14% of traumatic elbow injuries [1,2]. They rank fourth after supracondylar fractures, epitrochlear fractures and external condyle fractures [3]. There are two anatomical types of radial neck fracture: the most common is the metaphyseal fracture of the radial neck, followed by the epiphyseal separation fracture (Salter-Harris II) [4]. All ages are concerned, with a peak frequency around 9-10 years [5]. The mechanism is essentially indirect; they are common FOOSH injuries which are caused by having “fallen onto an out-stretched hand” [6]. Radial neck fracture in children is an intra-articular fracture on a growing bone. Its evolutionary risk can be at the origin of major anatomical and functional sequelae secondary to the development of a malunion or abnormalities of growth by attack of the growth cartilage, imposing a specific treatment. Various surgical techniques have been used to treat radial neck fractures in children, such as percutaneous joystick reduction with Kirschner wires and open reduction with or without internal fixation, but the technique of stabilization by elastic stable intramedullary pinning, first described by Metaizeau in 1980 and subsequently developed in 1993, significantly improved the results of surgery [7,8]. The principles of this technique are the respect of the biology of the bone consolidation by a percutaneous intramedullary fixation, the non-aggression of the physis thus avoiding the growth disorders, and the early functional recovery. The quality of the results means that this method is now accepted worldwide, its benefits being widely recognized [9]. The main objective of this case series is to assess the efficacy of Metaizeau technique in the treatment of pediatric radial neck fractures.

## Methods

**Figure 1:**
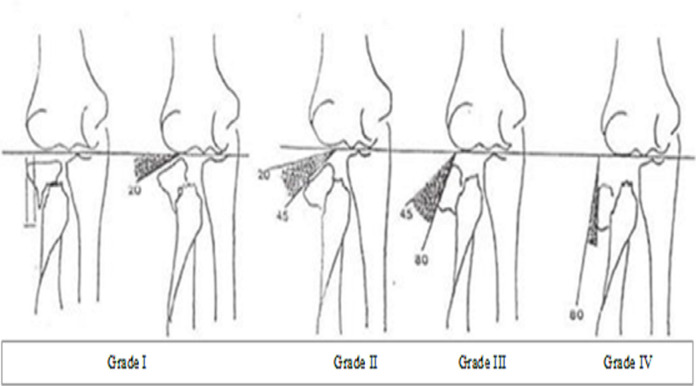
Metaizeau classification of radial neck fracture in children

**Figure 2:**
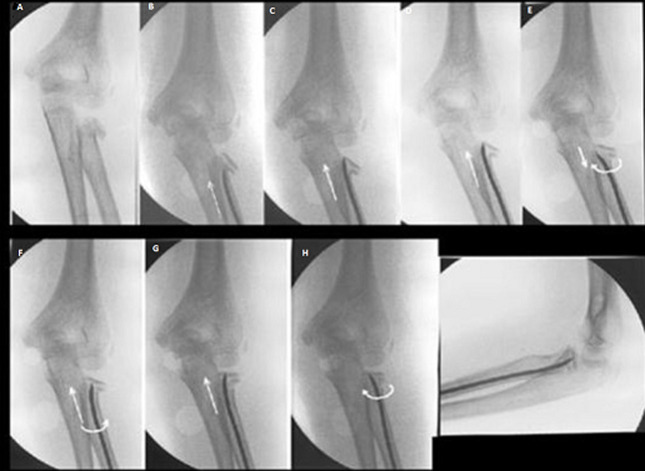
Metaizeau intramedullary nail technique

We retrospectively reviewed the clinical records and radiographs of all pediatric patients treated for displaced radial neck fractures at Sahloul University Hospital, from 2001 to 2017. Inclusion criteria were, all the patients with open growth plate of the proximal radius at the injury time, fracture tilt >20 degrees, with or without associated lesions and minimum follow-up of 6 months. Exclusion criteria were, open fracture, incomplete medical or radiographic records, orthopedic treatment and associated head or diaphyseal fracture. Fractures were classified according to Metaizeau classification [7] (Figure 1): grade 1: translation less than 3 millimeters or epiphysis tilt less than 20°; grade 2: tilt between 20° and 45°; grade 3: tilt between 45° and 80°; grade 4: more than 80° of epiphyseal tilt. All included patients were treated with the Métaizeau intramedullary nail technique (Figure 2).

**Surgical technique:** under general anesthesia, the upper limb is prepared and draped from the axillary fold to the hand and is placed on a hand table, using an image intensifier, an attempt for closed reduction was made by pulling the extended elbow in a varus direction and applying pressure on the lateral side of the elbow and performing repetitive supination-pronation of the elbow. Once the best position of the radial head fracture is obtained, we identified the lateral side of distal radial physis and made a 2cm incision. The soft tissues are dissected carefully, avoiding injury to the delicate cutaneous branch of the radial nerve. The cortex is perforated with a drill. A 1.2-2.0mm K-wire, guided by a handle, is introduced into the medullary canal and then hammered upward until its tip reached the displaced epiphysis. The K-wire was then pushed in order to elevate the epiphysis and then turned 180 degrees around its axis to relocate the radial head so the tip points medially reducing the fracture. If the reduction was still not satisfactory, a Kirschner wire was inserted percutaneously, through the fracture from the lateral side and used as a lever arm to reduce the fracture. Finally, the fracture was stabilized with one nail. We used post-operative immobilization in a long-arm cast for 3-4 weeks. The wire is removed after 2-3 months, when the fracture is completely healed.

Average follow-up was 13.5 months (range: 7 to 36 months). At final follow-up we evaluated clinical and radiographic results. The clinical evaluation included a full examination of the upper limb. The range of motion of the elbow (flexion, extension) and forearm (supination, pronation) was measured and compared to the contralateral side. Subjects were divided into 4 groups according to the Mayo elbow performance score (MEPS) [10] (Table 1). It is comprised of 4 items: pain, range of motion, stability and daily function [11]. The results are graded with a maximum of 100 points and categorized into 4 groups: excellent >90; good 75-89; fair 60-74; poor <60. Standardized anteroposterior and lateral elbow radiographs made at the time of the initial management, after 6 weeks (time of consolidation) and at time of the most recent follow-up (final follow-up), were available for every patient. MRI (magnetic resonance imaging) was performed when avascular necrosis of the radial head was suspected. Any residual angulation was measured on radiographs. Results were considered as follows: excellent, if the reduction was anatomic; good, if a simple shift or inclination not exceeding 20° persisted; fair, if the tilt was between 20° and 40°; and poor if it was beyond 40° or bone changes such us, avascular necrosis and nonunion. Infectious or neurologic (radial nerve injury) complications were investigated, as well as nonunion, avascular necrosis of the radial head or radio-ulnar synostosis.

**Table 1:** Mayo elbow performance score (MEPS)

Variable	Definition	Points
Pain (maximum 45 points)	None	45
	Mild	30
	Moderate	15
	Severe	0
Range of motion, degrees (maximum 20 points)	Arc >100	20
	Arc 50-100	15
	Arc <50	5
Stability (maximum 10 points)	Stable	10
	Moderately unstable	5
	Grossly unstable	0
Function (maximum 25 points)	Comb hair	5
	Feed onself	5
	Personal hygiene	5
	Put on shirt	5
	Put on shoes	5

## Results

**Figure 3:**
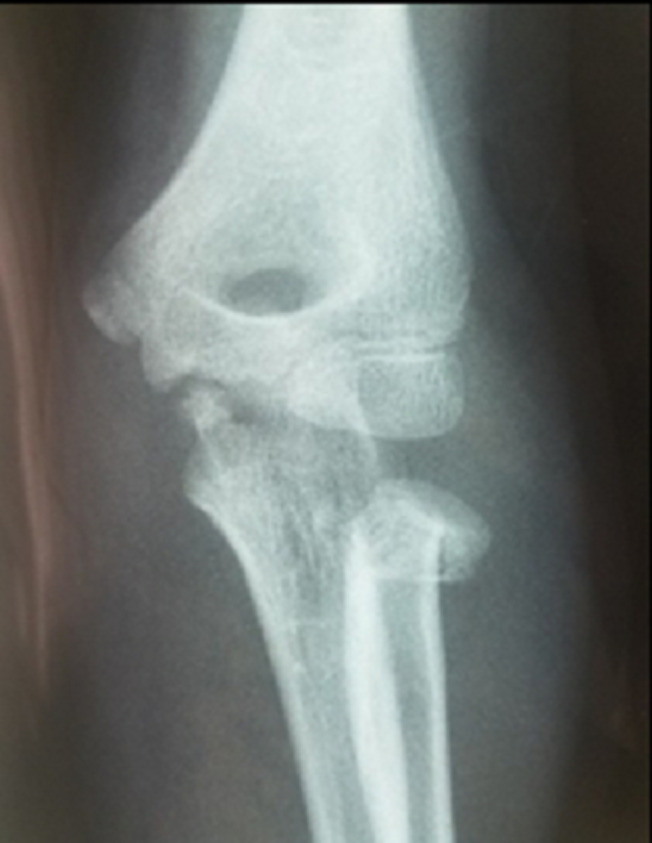
Metaphyseal fracture of the radial neck

**Figure 4:**
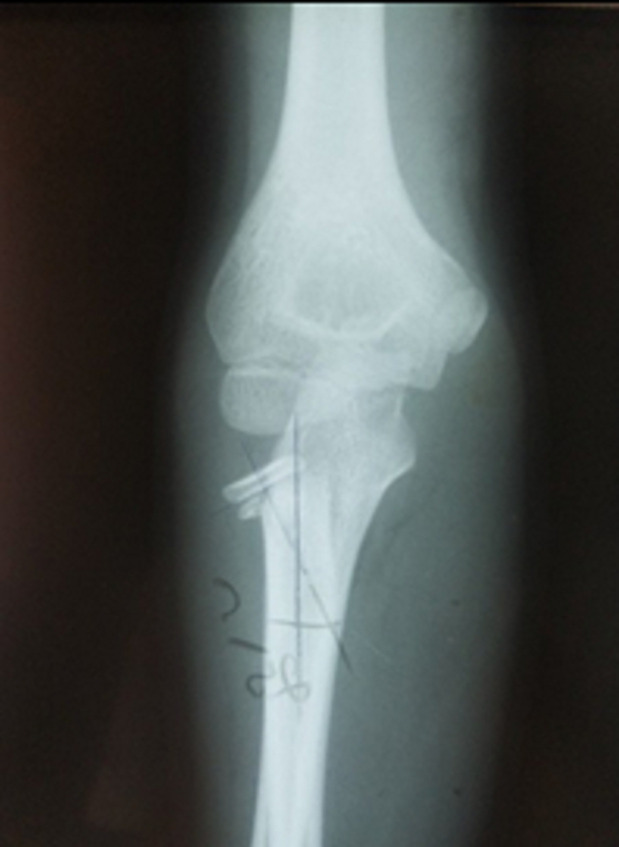
epiphyseal separation fracture type II of Salter and Harris

Twenty-two patients (11 males, 11 females) were included. Mean age at the time of fracture was 9 years (range 5 to 13y). The left elbow was the most often affected (12 of 22 fractures). The mechanism of radial neck fractures in our series was indirect in 14 cases, it was a valgus forced elbow following a fall on the outstretched hand with the elbow extended and the forearm supinated. A direct trauma following a fall with reception on the bending elbow was observed in 8 cases. Our series included a metaphyseal fracture of the radial neck in 8 cases (Figure 3) and an epiphyseal separation fracture type II of Salter and Harris in 14 cases (Figure 4). According to Métaizeau classification, we had 12 patients type II (54%), 7 patients type III (32%) and 3 patients type IV (14%). Seven patients had olecranon fracture of which 2 are associated with a posterior dislocation of the elbow. All patients were treated with Métaizeau technique. The lever arm technique was used in 3 cases and open reduction was necessary for 3 patients. Based on the MEPS score, we had 15 excellent, 2 good, 4 fair and 1 poor result. Sixteen patients had complete elbow mobility at last follow-up. Final radiographs showed 14 excellent, 4 good, 1 fair and 3 poor results. The socio-demographic and clinical data are summarized in Table 2. Different complications have been noted such as radial head necrosis in 1 case, pseudarthrosis in 1 case and peri-articular calcification in 2 cases. It was noted that epiphyseal fractures had a poor functional outcome in 4 cases whereas only 1 case of poor outcome in metaphyseal fractures. For type II fractures according to Métaizeau classification, all cases had excellent result and for type IV, we had poor result in all cases. Excellent results were seen in 78% of children who did not have a leverage reduction. Among patients requiring a surgical approach two had a poor result.

**Table 2:** socio-demographic and clinical data of the patients

Patient	Age (Year)	Sexe	Metaizeau classification	Technique	Follow up (month)	MEPS	Residual angulation (degree)
1	7	F	Grade III	Metaizeau	12	Excellent	0
2	9	M	Grade III	Metaizeau	7	Excellent	0
3	13	M	Grade II	Metaizeau	9	Excellent	0
4	5	M	Grade II	Metaizeau + Leverage	36	Good	5
5	10	F	Grade III	Metaizeau	9	Good	35
6	5	F	Grade II	Metaizeau	7	Excellent	0
7	5	F	Grade II	Metaizeau	8	Excellent	0
8	13	M	Grade II	Metaizeau	6	Excellent	0
9	7	F	Grade II	Metaizeau	12	Excellent	0
10	8	M	Grade II	Metaizeau + Leverage	12	Excellent	0
11	10	F	Grade IV	Metaizeau + Open surgery	36	Poor	90
12	13	M	Grade II	Metaizeau	6	Excellent	10
13	9	F	Grade IV	Metaizeau + Open surgery	6	Fair	40
14	11	M	Grade II	Metaizeau	10	Excellent	0
15	6	F	Grade II	Metaizeau	10	Excellent	0
16	9	F	Grade III	Metaizeau	7	Excellent	0
17	9	M	Grade IV	Metaizeau	8	Fair	5
18	8	F	Grade III	Metaizeau + Open surgery	6	Excellent	0
19	12	M	Grade II	Metaizeau	10	Excellent	0
20	9	M	Grade II	Metaizeau	24	Excellent	0
21	8	M	Grade	Metaizeau + Leverage	10	Fair	20
22	6	F	Grade	Metaizeau	24	Fair	5

## Discussion

Radial neck fracture in children is a rare event, accounting for only 1% of all pediatric fractures and approximately 5% of elbow fractures [1,12]. In the literature, the age interval for radial neck fracture is between 4 and 14 years with a maximum peak frequency between 9 and 12 years [13,14], this is explained by the significant fragility before complete ossification of the conjugal cartilage of the epiphysis (14-17 years old) [15]. In the Stiefel D series [16], the average age is 8 years and 4 months. This is the closest series to ours concerning the average age of patients. Referring to the literature, we see that the predominant mechanism in radial neck fracture in children is essentially indirect, is often caused by fall onto an outstretched hand with the elbow extended or slightly flexed and a valgus force applied to the elbow joint [17]. There is controversy regarding in which angle radial neck fracture can be managed conservatively or how much angulation should be operated. Most of the authors agree that more than 30° angulation require reduction and surgical treatment for children under the age of 10 and more than 15° at the end of growth [18]. However, the treatment strategy is up to surgeon and it is clear that younger patients have more chance to remodel [19]. Locke *et al*.reported that radial neck fractures with an angulation up to 50° in children under the age of 10 had good results with conservative treatment [20] whereas Al-Aubaidi *et al*.used surgical reduction and fixation in patients who had radial neck fractures with an angulation over 30° with excellent results [21].

There are several surgical options, described in the literature. They include percutaneous reduction with or without fixation and open surgery [19], which is generally reserved for large displacement fractures and more than 60° angulation [22]. Although Metaizeau [7] and PH. Tollet [23] believe that closed surgery may be indicated even in the case of a large displacement fracture. The open surgery allows an anatomical reduction of the fracture, but it compromises the epiphyseal vascularization which generates a high incidence of complications (radial head necrosis) [24] and a rate of bad result in 40% of the cases [5]. Metaizeau JP [25] proposed a technique of closed reduction and intramedullary pinning for radial neck fractures. A Kirschner wire is inserted from the posterolateral aspect of the radial neck with the forearm pronated to avoid injury to the posterior interosseous nerve. Reduction is achieved by turning around the nail 180°. Percutaneous leverage reduction is used for severely displaced fracture. In our series we associated leverage reduction in 3 cases. The results of our study and review of the literature did not objectify nerve complications related to intramedullary pinning, but some authors report that percutaneous leverage reduction involves a risk of damage to the motor branch of the radial nerve, lesions found by Pontailler [26] and Fasol [27].

In our series, late complications were noted in 5 patients. A child who had open surgery after failure of the Metaizeau technique, presented 3 types of complications which are radial head necrosis, pseudarthrosis and stiffness of the elbow. We also noted 2 cases of intra-articular calcifications and 2 cases of stiffness of the elbow (flessum). After a literature review, elevated rates of elbow stiffness and radial head necrosis have been noted after open surgery reduction [16]. The stiffness of the elbow after Metaizeau technique is seen especially in the forms treated late and in the case of associated lesion, in particular a dislocation of the elbow which constitutes an important prognostic factor. Some studies have noted that the prognosis was all the worse as the child was older [16,28]. Therefore, an incomplete reduction in fractures of the radius neck can be tolerated in younger children. Penneçot [4] and Tibone [29] have considered that metaphyseal fractures have a better prognosis than epiphyseal fractures. The surgical option used is one of the determining elements of the final functional result. It is clear that the Metaizeau technique reduced the need for open reduction and internal fixation [21].

It was also noted, according to the results of our series, that fractures whose reduction was considered good or average (residual angulation between 0° and 20°) can progress favorably towards an excellent radiological result. This has been reported by several authors [7,19]. In fact, Metaizeau [7] noted the absence of remodeling when the angulation exceeds 10° to 15° in children over 10 years old and 20° to 30° in small children. Limitation on this study are the small number of patients, which can be justified by the low prevalence of such cases and the fact of being retrospective missing randomization. Nevertheless, the results agreed with the literature which may contribute to the development of the technique. An additional study with higher methodological standards, including comparative study with other methods of treatment and larger sample size, is required to better confirm the efficacy of Metaizeau technique.

## Conclusion

The technique of closed reduction and distal intramedullary nail fixation of the radial neck fractures in children described by Metaizeau in 1980 changed the prognosis of radius neck fracture in children and has given in recent series better functional and anatomical results with fewer secondary and late complications. Despite the small number of patients in our series, we believe that flexible intramedullary nailing using the Metaizeau technique is a good option for displaced radial neck fractures in children.

### What is known about this topic

- Radial neck fracture in children can be at the origin of major anatomical and functional sequelae;
- Metaizeau technique reduced the need for open reduction and internal fixation.

### What this study adds

- Identify the main clinical features of radial neck fracture in children in Sousse, Tunisia;
- Assess the efficacy of Metaizeau technique in the treatment of pediatric radial neck fractures.
